# Obituary: Gail D. Adams, Ph.D. 1918–2010

**DOI:** 10.1120/jacmp.v11i4.3413

**Published:** 2010-07-22

Gail D. Adams was one of the founding members of both AAPM and ACMP. He helped shape the profession that all of us enjoy today. Gail had a major impact on scientific and professional activities. His pioneering efforts were felt in all areas of medical physics and radiation oncology. Gail was very clinical and he participated as a partner of the radiation oncologist in the radiation treatment process. He was equally proud of establishing an early educational program for medical physicists. Gail was active throughout his career in teaching, research, publications, mentoring, scientific and professional societies, and he participated in establishing national standards.

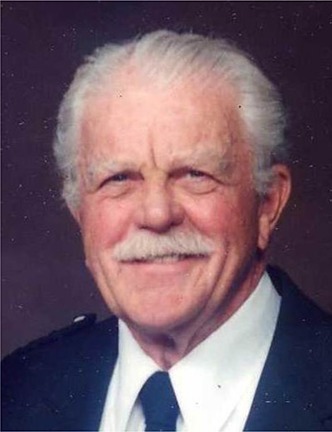

Gail is survived by his wife, Reba, three children, two step‐children and several grand‐children.

Gail was born and raised in Cleveland, OH. He received his B.S. degree from Case Institute of Technology in 1940. Gail received a stipend to attend graduate school at the University of Illinois where he was a Graduate Assistant to Donald Kerst, the inventor of the betatron. During these years, he was involved with research on the betatron, which included work for the government in support of the war effort. During the Manhattan Project, Gail served as a consultant due to his expertise on the betatron. At Los Alamos with Enrico Fermi, Gail worked to apply a single burst of X‐rays to see if it had any effect on the atomic bomb. It didn't, and the following year the bomb was dropped on Japan. After receiving his Ph.D. in 1943, Gail remained at the University of Illinois as an Assistant Professor. When Donald Kerst left the University, Gail was named Department Chairman; he remained at the University until 1951.

An unfortunate situation led Gail into the application of physics to medicine. In November 1947, a graduate student was diagnosed with a glioblastoma. Donald Kerst had made people aware that the betatron would also be useful for treating cancer. This encouraged the staff to consider treatment with the betatron. A radiologist agreed to be medically responsible for the student and the physics staff prepared the dosimetry. The autopsy showed the surgical defect had a boundary that radiation had cleaned up, but regional extension was still present. This made such an impact on Gail that he said that he wanted to “go some place to make a difference”.

Gail left the University of Illinois to accept a position as Research Physicist and Associate Director of Radiology at the University of California San Francisco. He worked with Dr. Stone on the development of a 70 MV synchrotron for radiation treatment.

In 1964, Gail accepted a position as Clinical Professor at the University of Oklahoma Health Sciences Center. During the interview, the University agreed to support Gail's plans to start a graduate program for medical physicists. The program started in 1970 with four students; by 1984, twenty eight had graduated. Dr Borgardus, Chair of Radiation Oncology, often referred to Gail as the backbone of clinical and educational programs at OUHSC. Gail arranged for the UCSF synchrotron to be relocated to Norman, OK, where it continued to be used as a research tool by many graduate students. Gail retired in 1984 as Professor and Vice‐Chairman.

During 1958 and 1959, Gail chaired a committee to look into establishing an organization for medical physicists. At that time, medical physicists had avenues for their scientific output through *Radiology* and *Radiation Research*, but had no structure for national recognition. Gail suggested the initial scope of the organization was to be professional. A Constitution was adopted in 1959, forming the AAPM. Gail, a Charter Member, was elected the first President of AAPM in 1960. After several years of scientific sessions at RSNA, the newly formed AAPM decided to have its own meeting that would include both scientific and professional sessions.

In recognition of Gail's pioneering efforts for AAPM, as well as his contributions toward educational, scientific and professional activities, he was elected AAPM Fellow and, in 1982, was awarded the William D. Coolidge Award. In addition, Gail was the first Editor of *Medical Physics*, serving from 1974–1978 (Volumes 1–5).

At the Annual Meeting in 1982, the AAPM Board approved funding to establish a Constituting Panel to look at forming a separate Professional College and Gail was elected Chair of the Panel. As a result of the Panel's recommendation, the ACMP was established. Gail was ACMP's first Chairman and served for two terms. He was later elected ACMP Fellow, and awarded the Marvin M.D. Williams Award in 1989.

Before Gail retired from the OUHSC, he and Reba built their retirement home in the mountains outside Pagosa Springs, CO. They lived there until moving to Talent, OR, in 1995.

During the 50^th^ AAPM Annual Meeting in Houston in 2008, Gail gave timely advice to young physicists: “Be dedicated to what you are doing. There is no answer for half‐way measures when patients' lives are in your hands.” Gail instilled this in all of his students; I first heard this when he was an advisor to my dissertation committee from 1970–1971.

Gail truly did make a difference, and was proud that medical physicists today continue to follow his lead to “make a difference”. Gail is described by his stepdaughter as “quiet and patient, hiding under an imposing exterior and solid competency”.

Alex Turner, Ph.D.

Breckenridge, CO

I would like to thank the ACMP staff for sharing background material, and especially Reba and her family for providing insight into the life of Gail Adams.

